# Acute Portal Vein Thrombosis Associated With Anastrozole Therapy: A Report of a Rare Case

**DOI:** 10.7759/cureus.101739

**Published:** 2026-01-17

**Authors:** Rim A Boutari, Perla E Abboud, Lea S Issa, Abed AlRaouf Kawtharani, Leonardo N Naffah, Antoine Abou Rached

**Affiliations:** 1 Gastroenterology and Hepatology, Lebanese University Faculty of Medical Sciences, Beirut, LBN; 2 Internal Medicine, Lebanese University Faculty of Medical Sciences, Beirut, LBN; 3 Radiology, Lebanese University Faculty of Medical Sciences, Beirut, LBN

**Keywords:** anastrozole, aromatase inhibitor, breast cancer, mesenteric vein thrombosis, portal vein thrombosis

## Abstract

Portal vein thrombosis (PVT) is a rare but potentially life-threatening condition that most often occurs in the setting of liver disease, malignancy, local inflammation, certain medications, or hypercoagulable disorders. We report a rare case of acute PVT in a 71-year-old woman with a history of breast cancer in remission who was receiving adjuvant anastrozole therapy and presented with abdominal pain. CT revealed extensive thrombosis involving the portal vein and superior mesenteric vein. The workup excluded malignancy recurrence and infection, revealing only heterozygous factor V Leiden. With no other clear cause identified, anastrozole was considered the most likely etiology. This case highlights the importance of recognizing aromatase inhibitors as potential contributors to venous thrombosis.

## Introduction

Portal vein thrombosis (PVT) is an uncommon diagnosis, with an estimated prevalence of approximately 1%. It may lead to serious complications, including mesenteric ischemia and portal hypertension [1]. Multiple risk factors have been described, including liver disease, malignancy, sepsis, certain medications, myeloproliferative disorders, and inherited or acquired prothrombotic conditions [2]. Malignancy-associated thrombosis is well recognized, with the highest risks occurring in pancreatic, lung, brain, and ovarian cancers. Breast and prostate cancers carry comparatively lower thrombotic risk [3].

Among medications, anti-angiogenic agents [4], immunomodulators [5], oral contraceptives, and selective estrogen receptor modulators (SERMs) [6] are well-documented culprits. Tamoxifen, in particular, increases thrombotic risk by altering procoagulant and anticoagulant factor synthesis and enhancing platelet adhesion [7]. In contrast, aromatase inhibitors such as anastrozole are associated with a significantly lower thrombotic risk profile compared with tamoxifen [8]. Proposed mechanisms for anastrozole-induced thrombosis include estrogen depletion leading to endothelial dysfunction [9].

Case reports implicating anastrozole in venous thrombosis remain exceedingly rare, with only one published case of mesenteric vein thrombosis temporally linked to aromatase inhibitor therapy [10]. Here, we present a second case of acute PVT in an elderly patient receiving anastrozole.

## Case presentation

A 71-year-old woman presented to our hospital with a one-week history of postprandial, diffuse abdominal pain. Her medical history was notable for type 2 diabetes mellitus, hypothyroidism, and metachronous bilateral breast cancer (two primary breast cancers diagnosed at different times). The first breast malignancy, diagnosed three years earlier in the right breast, was hormone receptor-positive, with an estrogen receptor score of 8 out of 8 and a progesterone receptor score of 7 out of 8 on the Allred scale. Human epidermal growth factor receptor 2 (HER2) was positive by immunohistochemistry (score 3 out of 3) and confirmed by fluorescence in situ hybridization.

One year later, an incidental follow-up imaging study identified a second lesion in the left breast, which was triple-negative, with estrogen receptor and progesterone receptor scores of 0 out of 8 and HER2 negative (score 0 out of 3). She subsequently underwent bilateral total mastectomy and completed one year of adjuvant trastuzumab therapy following the initial surgery.

At the time of presentation, she was maintained on oral anastrozole (1 mg daily), which she had been receiving for approximately three years as part of her adjuvant hormonal therapy. Her chronic medications also included Euthyrox (levothyroxine) for hypothyroidism and a basal-bolus insulin regimen for diabetes management. She had no personal or family history of venous thromboembolism (VTE) and no recent abdominal infection or surgery.

Three days prior to admission, she was evaluated in the outpatient clinic for similar abdominal pain. Laboratory tests at that time demonstrated a cholestatic pattern of liver enzyme elevation and a markedly elevated CRP (Table 1).

**Table 1:** Laboratory findings at outpatient evaluation (Day −3) and at admission (Day 0)

Parameter (unit)	Outpatient (Day -3)	Admission (Day 0)	Normal range
Hemoglobin (g/dL)	11.8	11.5	12-16 g/dL
Hematocrit (%)	33	32	36-46%
Platelets (×10⁹/L)	251	233	150-450 × 10⁹/L
Alkaline phosphatase (U/L)	907	874	44-147 U/L
Gamma-glutamyl transferase (U/L)	362	379	9-48 U/L
Aspartate aminotransferase (U/L)	60	51	10-40 U/L
Alanine aminotransferase (U/L)	46	38	7-56 U/L
Lipase (U/L)	17	-	13-60 U/L
CRP (mg/L)	298	271	<5 mg/L

An abdominal ultrasonography at that time showed a normally contoured gallbladder without stones but revealed a 38 × 32 mm hypoechoic, heterogeneous mass anterior to the pancreatic head and abutting the abdominal aorta. This finding raised concern for either a pancreatic lesion or a vascular abnormality.

Because her symptoms persisted, she presented again three days later (Day 0). On admission, she was hemodynamically stable with normal vital signs. Physical examination revealed mild abdominal distension and diffuse tenderness, most prominent in the epigastric and periumbilical regions. Repeat laboratory studies again demonstrated a cholestatic pattern of liver enzymes with normal bilirubin and a persistently elevated CRP, although the CRP was trending downward compared with the prior measurement (Table 1).

**Figure 1:**
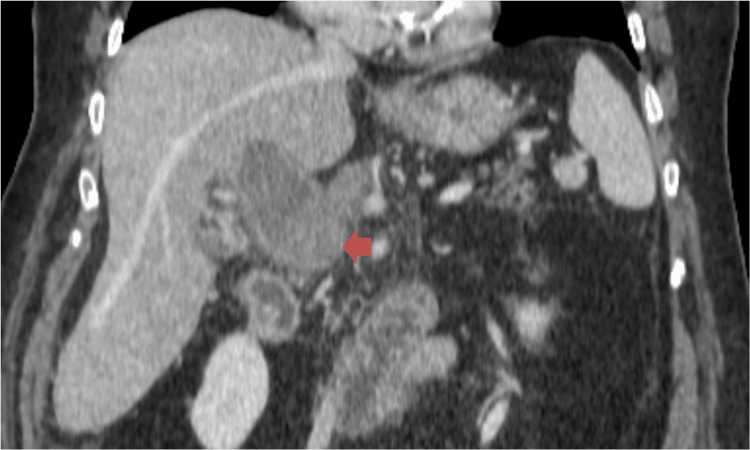
Coronal abdominal CT image highlighting the PVT (red arrow) PVT, portal vein thrombosis

**Figure 2:**
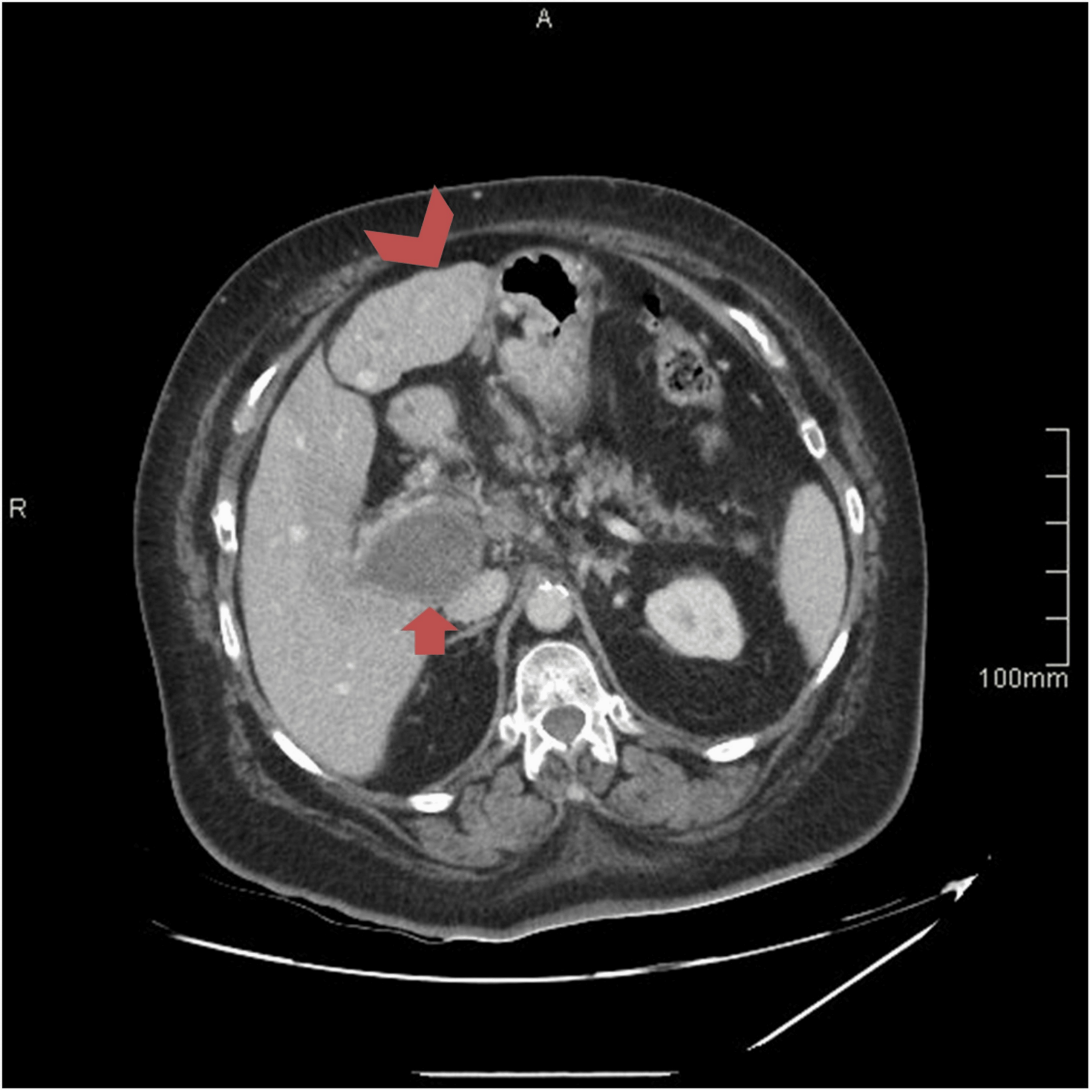
Axial abdominal CT image demonstrating PVT (red arrow) and a mildly lobulated liver contour (red arrowhead) PVT, portal vein thrombosis

A triphasic contrast-enhanced CT scan of the abdomen and pelvis revealed marked dilation of the portal vein, measuring up to 4 cm, with complete absence of contrast opacification, consistent with PVT. The thrombus appeared as spontaneously hyperdense, non-enhancing material and extended into the superior mesenteric vein and its branches, while the splenic vein remained patent. The liver and spleen were normal in size; however, the liver demonstrated a mildly lobulated contour (Figure 1, Figure 2).

The patient was managed conservatively with bowel rest (nothing by mouth) and intravenous hydration. Therapeutic anticoagulation with enoxaparin (1 mg/kg twice daily) was initiated. A hematology consultation was obtained, and tumor markers, along with an extensive thrombophilia workup, revealed a heterozygous factor V Leiden mutation (Table 2).

**Table 2:** Thrombophilia panel and tumor marker results CA, cancer antigen; CEA, carcinoembryonic antigen

Parameter/test	Result	Normal range
Factor V Leiden mutation	Heterozygous	No mutation detected
Protein C activity	110%	70-140%
Protein S activity	115%	60-130%
Prothrombin gene mutation	No mutation detected	No mutation detected
JAK2 mutation	No mutation detected	No mutation detected
Antithrombin III activity	89%	80-120%
Lupus anticoagulant	No mutation detected	No mutation detected
Anticardiolipin antibodies	No mutation detected	No mutation detected
β2-glycoprotein I antibodies	No mutation detected	No mutation detected
CA 19-9	27.7 U/mL	<37 U/mL
CA 15-3	23.6 U/mL	<30 U/mL
CEA	2 ng/mL	<5 ng/mL

A contrast-enhanced chest CT scan showed no evidence of breast cancer recurrence or metastasis. Over the subsequent days, her abdominal pain gradually improved, accompanied by declining CRP levels. An oral diet was reintroduced on hospital Day 3 and was well tolerated. She was discharged one week after admission in stable condition on a direct oral anticoagulant (rivaroxaban) for continued outpatient management.

In the absence of malignancy recurrence, infection, or other identifiable hypercoagulable factors, her PVT was attributed to a drug-induced cause, most likely the ongoing anastrozole therapy. Although exceedingly rare, this case highlights the potential for aromatase inhibitors to induce venous thrombotic events, including at atypical sites such as the portal venous system.

## Discussion

PVT is a relatively uncommon condition, with an estimated prevalence of approximately 1%, and can lead to serious complications such as mesenteric ischemia and portal hypertension [1]. The most frequently identified etiologies of PVT include liver disease, malignancy, local inflammatory processes, abdominal sepsis, and inherited or acquired prothrombotic disorders (e.g., antiphospholipid syndrome, hyperhomocysteinemia, factor V Leiden mutation, protein C or S deficiency, antithrombin III deficiency, and myeloproliferative disorders). Certain medications have also been implicated [2].

Extensive research has evaluated the association between malignancy and VTE. The highest VTE risks are observed in patients with brain, lung, pancreatic, and ovarian cancers, whereas breast and prostate cancers generally confer a lower risk [3]. Nevertheless, compared with age- and sex-matched controls without cancer, patients with breast cancer still demonstrate approximately a fourfold increased risk of thrombotic events [11]. More aggressive tumors with early metastatic spread are associated with even higher VTE rates [12].

Multiple medications have been reported as contributors to thrombosis, including antineoplastic agents with anti-angiogenic activity [4], immunomodulators [5], oral contraceptives, and hormone replacement therapy [6]. Drug-induced thrombosis can result from various mechanisms, such as direct endothelial injury leading to platelet adhesion (as seen with cytotoxic chemotherapy), increased blood viscosity following intravenous immunoglobulin therapy, and alterations in coagulation factor balance associated with oral contraceptives and SERMs [7,13]. For example, tamoxifen increases the synthesis of procoagulant factors (e.g., fibrinogen and prothrombin) and decreases physiological anticoagulants such as protein S, in addition to enhancing endothelial adhesion molecules, changes that collectively promote platelet aggregation [7].

Anastrozole is an aromatase inhibitor commonly recommended as adjuvant therapy for postmenopausal women with hormone receptor-positive breast cancer, typically for at least five years. Its most commonly reported adverse effects include decreased bone mineral density, arthralgias, and myalgias, which often contribute to therapy discontinuation, whereas thromboembolic events are relatively rare compared with those associated with SERMs [14,15]. The mechanism underlying anastrozole-associated thrombosis remains unclear; however, estrogen deprivation leading to endothelial dysfunction has been proposed [9].

This case illustrates a rare occurrence of PVT in a patient receiving anastrozole therapy. To our knowledge, it represents only the second reported instance of mesenteric or splanchnic venous thrombosis temporally associated with aromatase inhibitor use [10]. Potential alternative etiologies were thoroughly excluded through extensive laboratory evaluation and imaging, including normal cancer antigen (CA) 15-3 levels and no evidence of breast cancer recurrence, metastasis, or any other malignancy or infection.

Although our patient was heterozygous for factor V Leiden, which may confer an increased baseline risk of thrombosis, several considerations point to anastrozole as a likely precipitant. Her PVT developed after the initiation of anastrozole therapy in an elderly woman with no prior thrombotic events, no history of miscarriages, and no active cancer. These factors support a likely drug-related contribution to her thrombosis.

This case underscores the importance of maintaining a high index of suspicion for atypical thrombotic events in patients receiving aromatase inhibitors. Splanchnic venous thrombosis may represent an underrecognized adverse effect of anastrozole therapy. Reporting similar cases is essential to further clarify this association and to guide future strategies for risk assessment, prevention, and management.

## Conclusions

We report a rare case of PVT in a patient receiving anastrozole therapy. In the absence of cancer recurrence, infection, or other identifiable hypercoagulable factors, anastrozole appears to be the most likely causative agent. Clinicians should remain vigilant for the potential of rare thrombotic complications in patients treated with aromatase inhibitors. Additional case reports and larger studies are needed to further clarify this association, establish causality, and guide preventive and management strategies.
